# The association between frailty and the risk of mortality in critically ill congestive heart failure patients: findings from the MIMIC-IV database

**DOI:** 10.3389/fendo.2024.1424257

**Published:** 2024-08-05

**Authors:** Wenhua Shi, Hong Lin, Xinyu Zhang, Wenjing Xu, Taohua Lan, Wei Jiang, Xiankun Chen, Weihui Lu

**Affiliations:** ^1^ Second Clinical Medical College, Guangzhou University of Chinese Medicine, Guangzhou, Guangdong, China; ^2^ Department of Cardiology, The Second Affiliated Hospital of Guangzhou University of Chinese Medicine, Guangzhou, Guangdong, China; ^3^ State Key Laboratory of Dampness Syndrome of Chinese Medicine, The Second Affiliated Hospital of Guangzhou University of Chinese Medicine, Guangzhou, Guangdong, China; ^4^ Academician Chen Keji Workstation, The Second Affiliated Hospital of Guangzhou University of Chinese Medicine, Guangzhou, Guangdong, China; ^5^ Key Unit of Methodology in Clinical Research, The Second Affiliated Hospital of Guangzhou University of Chinese Medicine, Guangzhou, Guangdong, China; ^6^ Chinese Medicine Guangdong Laboratory, Hengqin, Guangdong, China; ^7^ State Key Laboratory of Traditional Chinese Medicine Syndrome, Guangdong Provincial Hospital of Chinese Medicine, Guangzhou University of Chinese Medicine, Guangzhou, Guangdong, China

**Keywords:** frailty, frailty index, elderly, congestive heart failure, MIMIC-IV database

## Abstract

**Background:**

Frailty is a severe, common co-morbidity associated with congestive heart failure (CHF). This retrospective cohort study assesses the association between frailty and the risk of mortality in critically ill CHF patients.

**Methods:**

Eligible patients with CHF from the Medical Information Base for Intensive Care IV database were retrospectively analyzed. The frailty index based on laboratory tests (FI_Lab) index was calculated using 33 variables to assess frailty status. The primary outcomes were in-hospital mortality and one-year mortality. The secondary outcomes were the incidence of acute kidney injury (AKI) and the administration of renal replacement therapy (RRT) in patients with concurrent AKI. Survival disparities among the FI_Lab subgroups were estimated with Kaplan-Meier survival analysis. The association between the FI_Lab index and mortality was examined with Cox proportional risk modeling.

**Results:**

A total of 3273 adult patients aged 18 years and older were enrolled in the study, with 1820 men and 1453 women included. The incidence rates of in-hospital mortality and one-year mortality rate were 0.96 per 1,000 person-days and 263.8 per 1,000 person-years, respectively. Multivariable regression analysis identified baseline FI_Lab > 0.45 as an independent risk factor predicting in-hospital mortality (odds ratio = 3.221, 95% CI 2.341–4.432, *p* < 0.001) and one-year mortality (hazard ratio=2.152, 95% CI: 1.730-2.678, *p* < 0.001). In terms of predicting mortality, adding FI_Lab to the six disease severity scores significantly improved the overall performance of the model (all *p* < 0.001).

**Conclusions:**

We established a positive correlation between the baseline FI_Lab and the likelihood of adverse outcomes in critical CHF patients. Given its potential as a reliable prognostic tool for such patients, further validation of FI_Lab across multiple centers is recommended for future research.

## 1 Introduction

Congestive heart failure (CHF) is a highly prevalent critical clinical condition. With the growth of aging populations, CHF has become a global public health challenge (1). Its prognosis is grim, particularly for those admitted to the intensive care unit (ICU). The in-hospital mortality rate for heart failure patients treated in cardiac intensive care units ranges from 4.2% - 12.5% (2, 3), and the 1-year survival rate after discharge ranges from 65.8%-74.5% (4, 5). Furthermore, these patients are often prone to concomitant acute kidney injury (AKI) which signals poor prognosis, underscoring the importance of early risk detection and stratification (6).

Older age is a significant risk factor for many serious diseases. Over time, people biologically accumulate molecular and cellular damage, including inflammation, genomic instability, and epigenetic changes (7, 8). These aging processes are closely linked to frailty. Aging contributes to reduced resistance to disease and external stressors (9). Longitudinal analyses have shown that frailty and multimorbidity vary with age, and that frail older adults affected by chronic or acute illnesses tend to experience more severe complications and have a higher risk of mortality (10, 11).

Frailty, characterized by diminished biological reserves and increased vulnerability to stress (12), has become more common with advancing age and the expansion of critical care services for older people (13). A meta-analysis has reported that frailty affects approximately 12% of the population (14). Additionally, mounting evidence has linked frailty to adverse outcomes in critically ill patients (15–17). Moreover, CHF patients who also experience frailty tend to have worse prognosis (18–20). Dewan et al. found that frailty, as assessed by the frailty index, was associated with worsening quality of life, and elevated hospitalization rates and mortality, in patients with heart failure with reduced ejection fraction (21).

Howlett et al. used a frailty index based on laboratory tests (FI_Lab) to identify community-dwelling older adults who are at increased risk of mortality (22). FI_Lab is more effective than other frailty assessment methods in predicting in-hospital mortality among critically ill patients (23). Moreover, FI_Lab has been identified as a potent predictor of both short- and long-term mortality in patients with critical acute myocardial infarction (24).

Numerous frailty screening and assessment tools have been employed on patients with CHF (25). However, the utilization of FI_Lab in the context of CHF remains uncharted territory. Therefore, this study assesses the association between frailty and the risk of mortality in critically ill patients with CHF by analyzing data from the Medical Information Mart for Intensive Care IV (MIMIC-IV) database.

## 2 Materials and methods

### 2.1 Study population

This study was a retrospective observational cohort study with data from the publicly available MIMIC-IV database (version 2.2). MIMIC-IV encompasses records from 73,181 ICU admissions at Beth Israel Deaconess Medical Center (Boston, MA, USA) between 2008 and 2019 (26, 27). The first author was granted access the database (Record ID: 56270677) (28, 29). The study was reported according to the STROBE guidelines (30). Any patients diagnosed with CHF were eligible for inclusion in this analysis. We employed both ICD9 and ICD10 codes for patient identification (**Supplementary Table 1**). In instances of multiple ICU admissions, we selected the record from the first admission. Exclusion criteria were: a) patients under the age of 18 years; b) an ICU stay shorter than 24 hours; c) the absence of essential data, including demographics, the six criticality scores, and the components needed to construct FI_Lab.

### 2.2 Data extraction

The baseline patient data were extracted from the MIMIC-IV database, including demographics, admission location, and severity assessments conducted within 24 hours of ICU admission. It included six severity assessments which covered a range of measures: sequential organ failure assessment (SOFA), logistic organ dysfunction system (LODS), systemic inflammatory response syndrome (SIRS), Oxford acute severity of illness score (OASIS), acute physiology score III (APS III), and simplified acute physiology score II (SAPS II), along with the Charlson comorbidity index (CCI). According to the Kidney Disease: Improving Global Outcomes (KDIGO) guidelines, AKI is defined by a ≥0.3 mg/dL rise in serum creatinine from baseline within 48 hours, an increase to 1.5 times the baseline level recorded during the preceding 7 days, or a urine output of less than 0.5 mL/kg/hour for 6 hours or more (31).

### 2.3 FI_Lab definition

To assess the frailty status, we developed FI_Lab score utilizing 33 test items sourced from the MIMIC-IV database. They encompassed data from 24 hours before and after the enrolled patients’ ICU admissions. In the present study, the actual FI_Lab range was determined to be 0.06 to 0.88, with a higher FI_Lab score indicative of a more severe degree of frailty. The ranges for the FI_Lab were non-frail (<0.36), pre-frail (0.36-0.45), and frail (>0.45), according to earlier investigations (22–24). We also analyzed the FI_Lab score as a continuous variable. The detailed construction methodology and specific items are explicated in the **Supplementary Materials** (**Supplementary Tables 1, 2**).

### 2.4 Clinical outcomes

The investigation began at the time that patients were admitted to the ICU. The primary outcomes of this study were patient mortality, encompassing both in-hospital and one-year mortality. The secondary outcomes were AKI incidence 24 hours after ICU admission and the administration of renal replacement therapy (RRT) in patients experiencing concurrent AKI.

### 2.5 Statistical analysis

Continuous variables were reported as mean ± standard deviation (SD) or median with interquartile range (IQR), whereas categorical variables were presented as counts and percentages (%). A t-test was employed for continuous variables with normal distributions, and a Kruskal-Wallis H-test was applied otherwise. Categorical variables were compared using the Pearson χ2 test.

Kaplan-Meier survival analysis was applied to determine the primary outcomes event incidence among distinct FI_Lab levels, with the log-rank test used to ascertain inter-group statistical significance. The assumption of proportional hazards in Cox analysis, verified through Kaplan-Meier curves, was met for 1-year mortality. A Cox regression model analyzed the relationship between FI_Lab and one-year mortality. In assessing the relationship between FI_Lab and 1-year mortality, we excluded patients who died during hospitalization. For secondary outcomes, we utilized the Fine-Gray subdistribution hazard model, which accounts for competing risks of death and expresses the associations as subdistribution hazard ratios (32). The cumulative incidence function curves was used to determine the incidence rates of secondary outcome events at different levels of FI_Lab. Logistic regression examined the association between FI_Lab and in-hospital mortality, along with the occurrence of AKI 24 hours after ICU admission. Adjustments for age, sex, ethnicity, admission location, SOFA score, and CCI were implemented in the multivariate models. The association between FI_Lab and clinical outcomes was further examined using restricted cubic spline regression models.

To ensure our results’ reliability, we performed two sensitivity analyses. APS III and SAPS II were adjusted for disease severity in multivariable regression models of FI_Lab with in-hospital mortality. We evaluated the effect of FI_Lab as a categorical variable in conjunction with the six disease severity scores to predict outcomes.

Subgroup analyses, categorized by sex, age group (≤65 and >65 years), obesity, diabetes, and hypertension were conducted to assess the uniformity in the prognostic influence of FI_Lab on the primary outcome. Additionally, we examined the interactions between FI_Lab and these stratified variables to identify any potential moderating effects.

The model’s predictive efficacy was evaluated using the area under the curve (AUC), integrated discrimination improvement (IDI), and net reclassification improvement (NRI). A DeLong test was applied to compare the AUC, both incorporating and excluding the FI_Lab score, to assess the incremental value of integrating FI_Lab as a continuous variable within the six disease severity scores in predicting adverse outcomes. We additionally reported the AUC (95% CI) for all laboratory variables in the adjusted model and used the DeLong test to compare the predictive accuracy of individual laboratory variables with that of FI_Lab.

All statistical analyses were executed using SPSS 18.0 for Windows (SPSS Inc., Chicago, IL, USA) and R (version 4.2.1), with a two-tailed *p*-value of <0.05 deemed statistically significant.

## 3 Results

### 3.1 Patient characteristics

**Figure 1:**
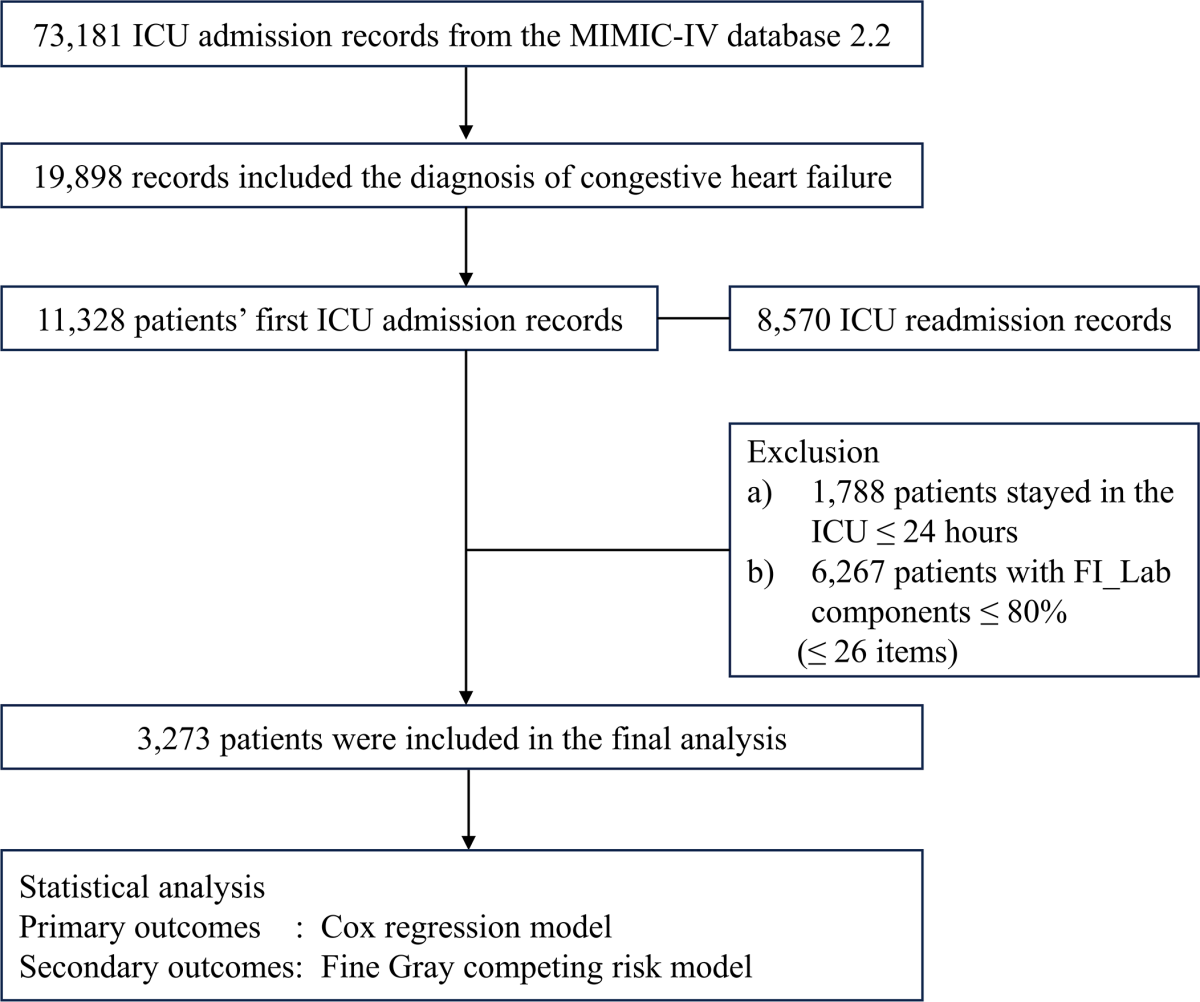
Flowchart of the detailed patient selection process.

Among the initial cohort of 11,328 patients presenting with CHF upon their inaugural admission to the ICU, our study ultimately encompassed 3,273 individuals who had surpassed a 24-hour ICU stay, and for whom there was adequate data to establish the FI_Lab (**Figure 1**). The demographic and clinical characteristics of the study population were stratified and described according to the frailty levels defined by FI_Lab. The non-frail patients were slightly older than the frail and pre-frail patients (median ages: 75.0, 74.9, 74.6 years, respectively, *p* < 0.05). There were no significant disparities in the sex distribution across the groups. Patients characterized as frail or pre-frail presented with a greater burden of comorbidities than non-frail patients (CCI points, 8, 8, 7, respectively, *p* < 0.001). Moreover, disease severity markedly differed, with frail patients exhibiting significantly higher SOFA scores than both pre-frail and non-frail groups (9, 6, 5, respectively, *p* < 0.001) (**Table 1**; **Supplementary Table 3**).

**Table 1:** Characteristics of included patients according to frailty status.

Variables	Total (n=3,273)	Non-frail (n=1,021)	Pre-frail (n=1,197)	Frail (n=1,055)	*P*-value
Demographics					
Age, years, median (Q1, Q3)	74.79 (64.28,83.25)	75 (64.65,84.67)	74.93 (64.52,83.21)	74.55 (63.67,82.12)	0.026
Sex, *n* (%)					0.208
Male	1,820 (56)	551 (54)	660 (55)	609 (58)	
Ethnicity, *n* (%)					0.004
White	2,164 (66%)	710 (70%)	803 (67%)	651 (62%)	
Black	288 (9%)	83 (8%)	103 (9%)	102 (10%)	
Other	821 (25%)	228 (22%)	291 (24%)	302 (29%)	
Admission location, *n* (%)					< 0.001
Emergency department	1,195 (37)	316 (31)	458 (38)	421 (40)	
Other hospitals	1,977 (60)	668 (65)	700 (58)	609 (58)	
Operating room	57 (2)	27 (3)	21 (2)	9 (1)	
Other	44 (1)	10 (1)	18 (2)	16 (2)	
Comorbidities					
Obesity, *n* (%)	507 (15)	136 (13)	206 (17)	165 (16)	0.041
Hypertension, *n* (%)	984 (30)	385 (38)	324 (27)	275 (26)	< 0.001
Diabetes, *n* (%)	1,313 (40)	337 (33)	511 (43)	465 (44)	< 0.001
CCI, points, median (Q1, Q3)	7 (6,9)	7 (5,8)	8 (6,9)	8 (6,10)	< 0.001
ICU admission					
SAPSII score, median (Q1, Q3)	41 (33,51)	35 (29,42)	41 (34,48)	50 (41,60)	< 0.001
SOFA score, median (Q1, Q3)	6 (4,9)	5 (3,7)	6 (4,9)	9 (6,13)	< 0.001
APSIII score, median (Q1, Q3)	53 (40,72)	39 (32,52)	51 (42,66)	72 (57,93)	< 0.001
LODS score, median (Q1, Q3)	6 (4,9)	4 (3,6)	6 (4,8)	8 (6,11)	< 0.001
OASIS score, median (Q1, Q3)	35 (28,42)	30 (26,37)	34 (28,41)	40 (34,48)	< 0.001
SIRS score, median (Q1, Q3)	3 (2,3)	3 (2,3)	3 (2,3)	3 (3,4)	< 0.001
Outcomes					
Hospital mortality, *n* (%)	559 (17)	63 (6)	153 (13)	343 (33)	< 0.001
1-year mortality, *n* (%)	716 (26)	165 (17)	289 (28)	262 (37)	< 0.001
Incidence of AKI ^a^, *n* (%)	1,573 (48)	445 (44)	564 (47)	564 (53)	< 0.001
RRT ^b^, *n* (%)	346 (11)	22 (2)	96 (8)	228 (22)	< 0.001

a AKI was defined as occurring 24 hours after admission to the ICU according to KDIGO guidelines.

b RRT, renal replacement therapy used in patients with AKI; ICU, intensive care unit; CCI, Charlson Comorbidity Index; SAPS II, simplified acute physiological score II; SOFA, sequential organ failure assessment; APS III, acute physiology score III; LODS, logistic organ dysfunction system; OASIS, Oxford acute severity of illness; SIRS, systemic inflammatory response syndrome.

### 3.2 The association between FI_Lab and primary outcomes

**Figure 2:**
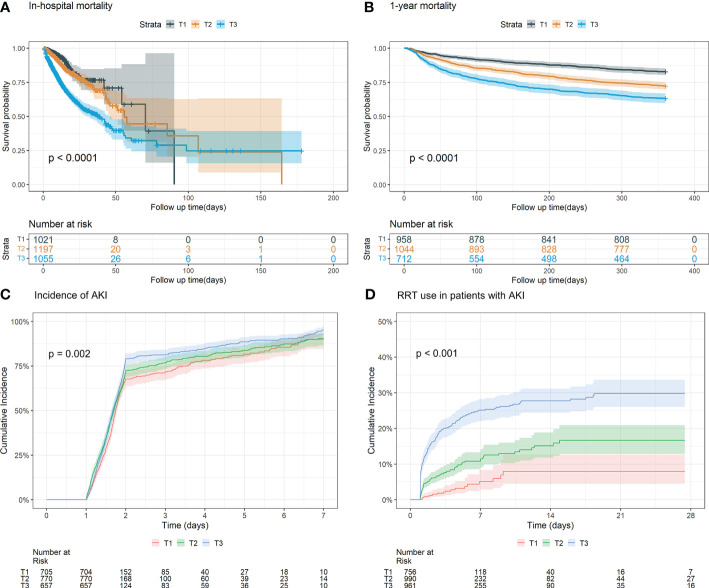
Kaplan-Meier survival analysis curves and cumulative event rate curves for the adverse outcomes. **(A)** Kaplan-Meier survival analysis curves for the in-hospital mortality of the whole study population. **(B)** Kaplan–Meier survival analysis curves for all-cause mortality according to groups at 1 year. **(C)** Cumulative incidence function curves for the incidence of AKI incidence after 24 hours of after ICU admission. **(D)** Cumulative incidence function curve of for RRT use in AKI patients. (FI_Lab tertile T1: 0.06–0.36; T2: 0.36–0.45; T3: 0.45-0.88).

**Figure 3:**
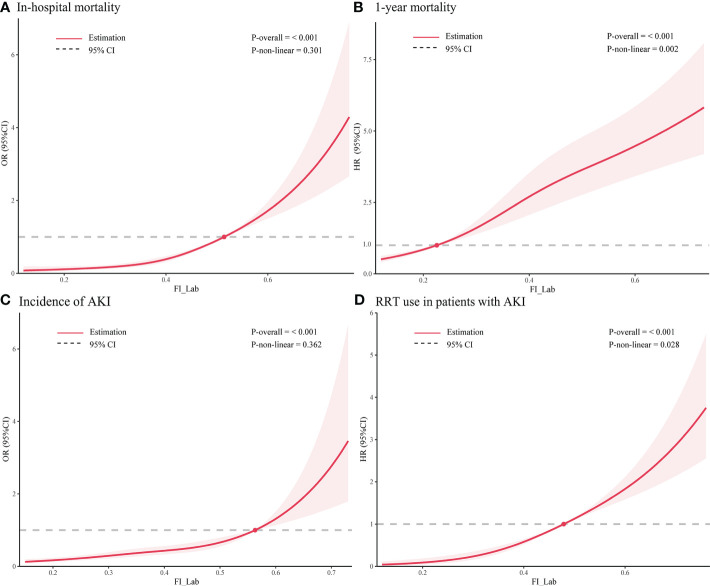
Restricted cubic spline regression analysis of adverse outcomes. Restricted cubic spline regression analysis of FI_Lab as a continuous variable with **(A)** in-hospital mortality and **(B)** 1-year mortality; spline curves showing the association between **(C)** FI_Lab and AKI incidence 24 hours after ICU admission and **(D)** RRT use in AKI patients. The solid red line represents the estimated adjusted odds ratio or hazard ratio, and the shaded band represents the 95% confidence interval. The dashed lines indicate an odds ratio or hazard ratio of 1.0. The spline curves were adjusted for age and sex.

**Figure 4:**
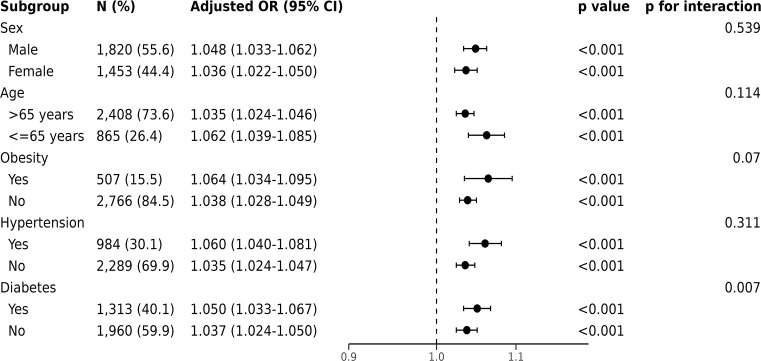
Forest plots of the odds ratios for in-hospital mortality by subgroup. The odds ratio (OR) was adjusted for age, sex, ethnicity, admission location, SOFA score and CCI points.

For in-hospital mortality, the Kaplan-Meier survival curves reveal a statistically significant difference across the FI_Lab groups (non-frail: 6.1%; pre-frail: 12.7%; frail: 32.5%, log-rank test *p* < 0.001, **Figure 2A**). Additionally, FI_Lab exhibited a significant association with in-hospital mortality within a multivariable model, both as a continuous variable (per 0.01-score increase: odds ratio (OR)=1.042, 95% confidential interval (CI):1.032-1.052; **Figure 3A**), and as a categorical variable (pre-frail: OR=1.58, 95% CI: 1.148-2.174; frail: OR=3.221, 95% CI 2.341–4.432; **Table 2**). Subgroup analyses revealed that the association between frailty and in-hospital mortality was more pronounced in diabetic patients than in non-diabetic patients (*p*-interaction<0.01, **Figure 4**).

**Table 2:** Association of FI_Lab with in-hospital and 1-year mortality.

Categories	Events (%)	Model 1	Model 2	Model 3
In-hospital mortality		OR (95%CI)	OR (95%CI)	OR (95%CI)
Continuous variable (per 0.01-score)		1.071 (1.062-1.08)	1.074 (1.066-1.083)	1.042 (1.032-1.052)
Categorical variable				
Non-frail	63 (6.1)	Ref.	Ref.	Ref.
Pre-frail	153 (12.7)	2.229 (1.641-3.027)	2.268 (1.668-3.084)	1.580 (1.148-2.174)
Frail	343 (32.5)	7.326 (5.505-9.747)	7.848 (5.882-10.471)	3.221 (2.341-4.432)
*P* for trend		<.001	<.001	<.001
1-year mortality		HR (95%CI)	HR (95%CI)	HR (95%CI)
Continuous variable (per 0.01-score)		1.031 (1.025-1.037)	1.035 (1.028-1.041)	1.03 (1.023-1.037)
Categorical variable				
Non-frail	165 (17.2)	Ref.	Ref.	Ref.
Pre-frail	289 (27.6)	1.712 (1.414-2.073)	1.742 (1.438-2.111)	1.495 (1.228-1.821)
Frail	262 (36.7)	2.479 (2.040-3.012)	2.660 (2.184-3.240)	2.152 (1.730-2.678)
*P* for trend		<.001	<.001	<.001

Model 1: unadjusted.

Model 2: adjusted for age, sex, ethnicity and admission location.

Model 3: adjusted for age, sex, ethnicity, admission location, SOFA scores and CCI points.

FI_Lab, frailty index based on physiological and laboratory tests; OR, odds ratio; HR, hazard ratio; SOFA, sequential organ failure assessment; CCI, Charlson comorbidity index; CI, confidential interval.

For 1-year mortality, elevated FI_Lab scores were associated with higher 1-year mortality (non-frail: 17.2%; pre-frail: 27.6%; frail: 36.7%; log-rank test *p* < 0.001, **Figure 2B**). After adjusting for multiple confounding factors, patients with elevated FI_Lab exhibited an increased risk of 1-year mortality (per 0.01-score: hazard ratio (HR) = 1.03, 95% CI: 1.023-1.038; **Figure 3B**). Moreover, pre-frail and frail patients demonstrated significantly elevated risks of 1-year mortality in comparison to non-frail individuals (HR=1.495, 95% CI: 1.228-1.821 and HR=2.152, 95% CI: 1.730-2.678, respectively; **Table 2**). Subgroup analyses revealed a heightened risk of 1-year mortality among frail patients over 65 years of age and those without obesity (*p*-interaction < 0.05, **Supplementary Figure 1**).

### 3.3 The association between FI_Lab and secondary outcomes

The cumulative incidence function curves demonstrate a significant disparity in the likelihood of AKI occurrence 24 hours after ICU admission between frail and pre-frail patients compared to non-frail patients (*p* = 0.002, **Figure 2C**). After accounting for the competing risk of death, frail and pre-frail patients with concomitant AKI exhibited a heightened risk of subsequent RRT utilization compared to non-frail patients (*p* < 0.001, **Figure 2D**). In the fully adjusted model, frailty was associated with the occurrence of AKI (OR=1.403, 95% CI: 1.031-1.909). There was also a significant correlation between frailty and increased utilization of RRT (subdistribution HR=2.89, 95% CI: 1.79-4.65) (**Supplementary Table 4**). After adjusting for both sex and age, the restricted cubic spline regression models demonstrated a dose-response relationship between FI_Lab and the incidence of AKI, as well as the utilization of RRT (*p*-nonlinear=0.362 and *p*-nonlinear=0.028, **Figures 3C, D**).

### 3.4 Sensitivity analyses

Sensitivity analyses indicate our findings’ robustness. Multivariable regression models, adjusted for APS III and SAPS II, affirmed the continued significance of FI_Lab’s association with in-hospital mortality. The added predictive value of FI_Lab, whether used as a categorical variable in combination with the 6 scores or as a continuous variable, remained consistent (**Supplementary Tables 5, 6**).

### 3.5 FI_Lab’s prognostic influence on the clinical outcomes

For the primary outcomes, the APS III scores exhibited the highest discriminatory capacity for in-hospital mortality (AUC = 0.80, 95% CI 0.78-0.82). Incorporating the FI_Lab into the APS III score improved its ability to identify individuals who had died in the hospital (ΔAUC = 0.008, *p* < 0.01), yielding an IDI of 0.012 (95% CI 0.006–0.017) and an NRI of 0.272 (95% CI 0.181–0.362). Moreover, the SAPS II score demonstrated superior discriminatory ability for 1-year mortality (AUC = 0.64, 95% CI 0.61-0.66). The incorporation of the FI_Lab further improved the SAPS II score’s capacity to differentiate patients with 1-year mortality (ΔAUC = 0.015, *p* < 0.05). This adjustment resulted in an IDI of 0.014 (95% CI 0.009 - 0.018) and an NRI of 0.28 (95% CI 0.196 - 0.365) (**Supplementary Table 7**).

For the secondary outcomes, the OASIS score exhibited the best predictive capability for AKI occurrence (AUC = 0.75, 95% CI 0.73-0.77). The incorporation of FI_Lab into the OASIS score did not significantly improve its predictive ability (ΔAUC=0.005, *p* = 0.145). We observed that the SOFA score could effectively distinguish the use of RRT in CHF patients with AKI (AUC=0.80, 95% CI 0.77-0.82). Furthermore, FI_Lab contributed to the improved discriminatory and reclassification performance of the SOFA score for RRT utilization (ΔAUC=0.015, *p* < 0.001; NRI=0.302, 95% CI 0.19-0.414) (**Supplementary Table 7**). In addition, the AUC for all laboratory variables included in the FI_Lab, as presented in the adjusted model, is detailed in **Supplementary Tables 8, 9**. The results indicate that FI_Lab is more accurate than each individual variable, with the difference being statistically significant (*p* < 0.001).

## 4 Discussion

In this retrospective cohort study of 3,273 patients, we demonstrated for the first time that FI_Lab is independently associated with both in-hospital and one-year mortality in critically ill CHF patients. After controlling for the competing risk of death, our study revealed a significant correlation between FI_Lab and the development of AKI 24 hours after ICU admission and progression to the need for RRT in CHF patients with comorbid AKI. Furthermore, our study showed that FI_Lab can better identify the increased risk of in-hospital mortality, 1-year mortality, and progression of AKI to the use of RRT in critically ill patients with CHF based on traditional disease severity scores.

The association between frailty and the prognosis in patients with CHF has been investigated in several studies. A study that screened 467 patients with chronic CHF using the clinical frailty scale showed that patients with frailty had more severe symptoms, higher N-terminal pro–B-type natriuretic peptide levels, and more comorbidity (33). This aligns with our study’s findings, indicating that frailty status in critical ill patients with CHF was associated with clinical outcomes. Vidán et al. assessed 450 hospitalized CHF patients according to Fried’s phenotype and found that 1-year mortality was higher in frail patients (HR=2.13, 95% CI 1.07-4.23) (34). In our study, the HR was higher than that reported by Vidán et al. (2.15 vs. 2.13). This discrepancy may have been attributed to differences in patient populations, as our study focused on critically ill patients, while Vidán et al.’s study did not. A meta-analysis found a significant difference in HRs for all-cause mortality in CHF patients when comparing studies using Fried’s phenotype to all included studies (1.80 vs. 1.54) (35). Weight loss and weakness in the Fried’s phenotype often characterize advanced CHF cachexia, potentially leading to overestimated mortality risk. However, the FI_Lab in this study more accurately reflects the decline in patients’ biological reserves, providing frailty assessments that are more reflective of real-world conditions.

Frail patients have a higher likelihood of developing renal insufficiency. Research has demonstrated that frailty is associated with the development of AKI in critically ill patients, with one in four critically ill patients who develop AKI being identified as frail at baseline (36). The clinical frailty scale has identified baseline frailty as an independent predictor of 90-day mortality in critically ill older patients with AKI (37). In our study, we observed that the occurrence of AKI 24 hours after ICU admission was independently associated with frailty in critically ill CHF patients. Another study utilizing the frailty index revealed that a higher percentage of frail chronic kidney disease patients required dialysis than non-frail chronic kidney disease patients (38). Our findings show that FI_Lab improves the identification of the risk of using RRT in patients with AKI. This suggests that frailty may be an important risk factor for disease progression in critically ill CHF patients with comorbid AKI.

Subgroup analysis suggested that FI_Lab is not significantly influenced by age, sex, or underlying diseases. Originally developed for older people in residential communities (22), FI_Lab in this study still holds prognostic value for patients under 65 years of age, with their frailty being associated with high in-hospital mortality and 1-year mortality risk. Interestingly, our research found that frailty in patients with underlying conditions such as hypertension, diabetes, and obesity was also correlated with overall mortality risk, indicating the potential utility of FI_Lab for patients with diverse underlying diseases. Furthermore, since we used FI_Lab in this study, we did not need to use cardiovascular disease-specific tests, simplifying the assessment of frailty in critically ill patients with CHF in practice.

Frailty increases the risk of mortality in patients with diabetes and should be given attention in the management of critically ill patients. Our study found a significant interaction between frailty and diabetes regarding in-hospital mortality in subgroup analyses. CHF patients often have complex conditions, and factors such as glucose fluctuations, malnutrition, and immunosuppression caused by diabetes may exacerbate the adverse effects of frailty. Multiple cohort studies have found that frailty is associated with an increased risk of cardiovascular disease and all-cause mortality in diabetic patients (39–41). Qin et al. discovered that a higher frailty index in individuals with type 2 diabetes is related to an increased risk of CHF (41). These clinical findings further support the complex interplay between diabetes and frailty. Our results indicate that an elevated frailty index has a more pronounced negative impact on critical ill patients with CHF and diabetes, thereby increasing their risk of in-hospital mortality. Therefore, strengthening the measurement and management of frailty in diabetes may help reduce the burden and mortality of critically ill CHF patients.

FI_Lab has the potential to improve predictive models’ accuracy. Our study revealed that FI_Lab significantly improved APS III and SAPS II score’s discriminatory ability for in-hospital mortality and 1-year mortality risk in critically ill CHF patients. However, it is important to note that gait speed, grip strength, and other physical functions are also important objective indicators for assessing frailty (42, 43), but they are challenging to measure in the ICU environment. Therefore, the use of FI_Lab can facilitate rapid identification of CHF patients who are at risk of mortality. Timely comprehensive management is crucial for frail patients; for instance, the use of multi-drug therapy as recommended in guidelines may increase the risk of adverse events, and should be avoided in cases of over-treatment. For frail patients, systemic interventions, such as exercise rehabilitation, dietary adjustments, and nutritional support, can be employed to improve clinical outcomes (20).

### 4.1 Limitations

This study’s limitations should not be overlooked. Firstly, our inclusion of patients with critical heart failure admitted to the ICU raises the possibility that FI_Lab may have been influenced by acute illness. Thus, the use of FI_Lab might not have entirely prevented the interpretation of disease severity rather than sole assessment of frailty in certain patients. Secondly, the lack of a uniform consensus on the diagnostic threshold for frailty with FI_Lab led us to define non-frail, pre-frail, and frailty using FI_Lab tertiles. Thirdly, the absence of information on cardiac function class and left ventricular ejection fraction in the MIMIC database, which are crucial for patients with CHF, hindered further exploration. Lastly, while the results of the sensitivity analysis demonstrated our findings’ robustness, it is important to note that our study was a retrospective single-center study. Therefore, the findings require further validation through prospective multicenter studies.

## 5 Conclusions

FI_Lab is correlated with an increased risk of in-hospital and 1-year mortality in critically ill patients with CHF. Incorporating FI_Lab into existing disease severity scoring systems could potentially refine the accuracy of mortality prediction. Additionally, prospective multicenter studies are needed to confirm the utility of FI_Lab in monitoring and managing CHF patients.

## Data availability statement

Publicly available datasets were analyzed in this study. This data can be found here: https://doi.org/10.13026/6mm1-ek67.

## Ethics statement

Ethical approval was not required for the study involving humans in accordance with the local legislation and institutional requirements. Written informed consent to participate in this study was not required from the participants or the participants’ legal guardians/next of kin in accordance with the national legislation and the institutional requirements.

## Author contributions

WS: Conceptualization, Data curation, Formal analysis, Writing – original draft. HL: Data curation, Methodology, Software, Writing – original draft. XZ: Data curation, Formal analysis, Writing – original draft. WX: Formal analysis, Methodology, Writing – original draft. TL: Writing – review & editing. WJ: Writing – review & editing. XC: Funding acquisition, Supervision, Writing – review & editing. WL: Funding acquisition, Supervision, Writing – review & editing.
