# Supplementary material for: The association between frailty and the risk of mortality in critically ill congestive heart failure patients: findings from the MIMIC-IV database
Source: Front Endocrinol (Lausanne). 2024 Aug 5;15:1424257. doi: 10.3389/fendo.2024.1424257 (PMC11330805; doi:10.3389/fendo.2024.1424257)
Supplement: Supplementary file 1 [file DataSheet_1.docx]

Supplementary Material

# Supplementary Figures and Tables

## Supplementary Figures


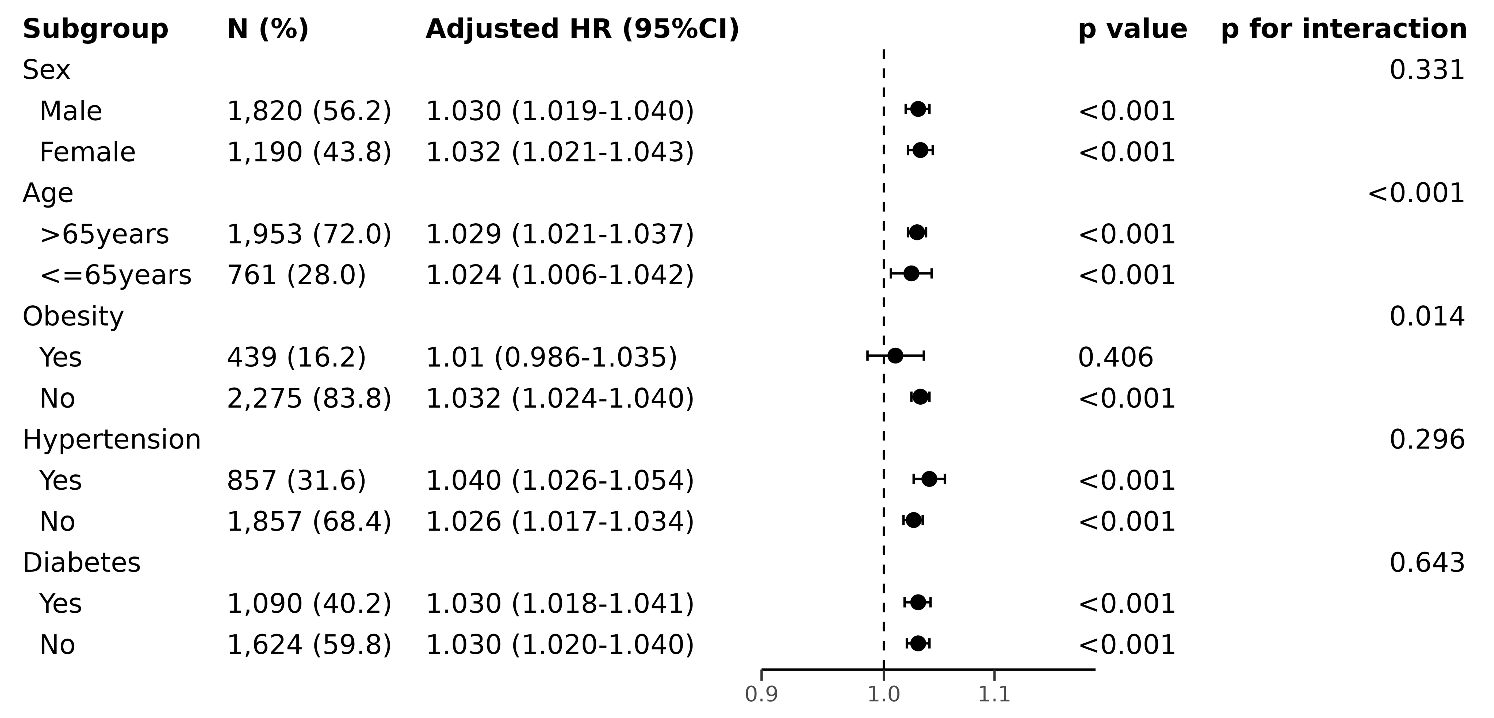


**Supplementary Figure 1.** Forest plots of hazard ratios for 1-year mortality by subgroup. The hazard ratio (HR) was adjusted for age, sex, ethnicity, admission location, SOFA score and CCI points.

## Supplementary Tables

### Supplementary Table 1. ICD codes used for congestive heart failure and detailed method for constructing FI_Lab

| Congestive heart failure | **ICD 10 codes:** I110 OR I130 OR I132 OR I50 OR I502 OR I5020  OR I5021 OR I5022 OR I5023 OR I503 OR I5030 OR I5031 OR I5032 OR I5033 OR I504 OR I5040 OR I5041 OR I5042 OR I5043 OR I508 OR I5082 OR I5083 OR I5084 OR I5089 OR I509.  **ICD 9 codes:** 40201 OR 40211 OR 40291 OR 40401 OR 40403 OR 40411 OR 40413 OR 40491 OR 40493 OR 4280 OR 42820 OR 42821 OR 42822 OR 42823 OR 42830 OR 42831 OR 42832 OR 42833 OR 42840 OR 42841 OR 42842 OR 42843 OR 4289 |
| --- | --- |
| FI_Lab | **Detailed construction method:** the FI_Lab was constructed using 33 items detected in the MIMIC-IV database of patients included in the study within 24 hours, before and after ICU admission. Among them, 30 items were obtained from venous blood samples, arterial blood gas samples, urine samples, and 3 vital signs: systolic blood pressure, diastolic blood pressure, and heart rate. If a test item was measured more than once, its mean value was used. The normal reference range provided in the database was used to judge each item. Items outside the range of the reference values were recorded as "1" (indicating defects). The FI_Lab score was calculated by summing the defects present and dividing by the total number of items. FI_Lab is theoretically between 0 and 1. In this study, the actual range was 0.06 to 0.88. |

FI_Lab: frailty index based on physiological and laboratory tests; ICD-10: International Statistical Classification of Diseases and Related Health Problems, Tenth Revision. ICU: intensive care unit.

### Supplementary Table 2. Reference range and the deficit proportion used to construct FI_Lab

| **Items** | **Reference range** | **3,273 patients, mean (SD) /median (Q1, Q3)** | **Deficit proportion, *N* (%)** |
| --- | --- | --- | --- |
| **Vital signs** |  |  |  |
| Systolic blood pressure (mm Hg) | 90–140 | 114.7±16.1 | 344 (10.5%) |
| Diastolic blood pressure (mm Hg) | 60–90 | 60.0±10.9 | 1,794 (54.8%) |
| Heart rate (bpm) | 60–99 | 85.8±16.4 | 747 (22.8%) |
| **Venous blood samples** |  |  |  |
| White cell count (×10^3^/μL) | 4–11 | 11.7 (8.6-15.6) | 1,867 (57.0%) |
| Platelet count (×10^9^/L) | 150–440 | 188.0 (136.8-251.7) | 1,179 (36.0%) |
| Hemoglobin (g/dL) | Female: 12–16 Male: 14–18 | 10.6±2.0 | 2,880 (87.9%) |
| Red blood cell distribution width (%) | 10.5-15.5 | 15.1 (14.0-16.7) | 1,331 (40.6%) |
| Total bilirubin (mg/dL) | 0–1.5 | 0.67 (0.4-1.2) | 496 (15.1%) |
| Alanine transaminase (units/L) | 0–40 | 26.0 (16.0-58.0) | 985 (30.0%) |
| Albumin (g/dL) | 3.5–5 | 3.4±0.6 | 1,318 (40.2%) |
| Alkaline phosphatase (units/L) | Female: 35-105  Male: 40-130 | 85.0 (64.0-119.0) | 818 (24.9%) |
| Lactate dehydrogenase (units/L) | 94–250 | 302.0 (224.0-462.0) | 1,295 (39.5%) |
| Urea nitrogen (mg/dL) | 6–20 | 28.5 (18.8-45.5) | 2,331 (71.2%) |
| Creatinine (mg/dL) | Female: 0.4–1.1 Male: 0.5-1.2 | 1.3 (1.0-2.0) | 1,905 (58.2%) |
| Glucose (mg/dL) | 70–110 | 134.5 (112.0-173.0) | 2,536 (77.4%) |
| Potassium (mmol/L) | 3.5–5.4 | 4.3±0.6 | 333 (10.1%) |
| Sodium (mmol/L) | 133–145 | 138.0±5.0 | 555 (16.9%) |
| Calcium (mg/dL) | 8.4–10.3 | 8.4 (7.9-8.8) | 1,615 (49.3%) |
| Phosphorus (mg/dL) | 2.7–4.5 | 3.8 (3.2-4.6) | 1,209 (36.9%) |
| Prothrombin time (s) | 9.4–12.5 | 14.6 (12.9-17.9) | 2,595 (79.2%) |
| International normalized ratio | 0.9–1.1 | 1.3 (1.2-1.6) | 2,648 (80.9%) |
| APTT (s) | 25–35 | 34.9 (29.3-48.8) | 1,793 (54.7%) |
| Fibrinogen (mg/dL) | 150–400 | 268.0 (198.0-397.0) | 402 (12.2%) |
| **Arterial blood gas samples** |  |  |  |
| PH | 7.35–7.45 | 7.4±0.1 | 1,219 (37.2%) |
| PO_2_ (mm Hg) | 85–105 | 123.6 (87.0-197.9) | 2,281 (69.6%) |
| PCO_2_ (mm Hg) | 35–45 | 40.0 (35.5-45.3) | 1,273 (38.8%) |
| Lactate (mmol/L) | 0.5–2 | 1.8 (1.3-2.7) | 1,029 (31.4%) |
| **Urine sample** |  |  |  |
| Leucocytes | Negative | 1,294 (39.5%) | 1,294 (39.5%) |
| Erythrocytes | Negative | 1,294 (39.5%) | 1,294 (39.5%) |
| Protein | Negative | 2,227 (68.0%) | 2,227 (68.0%) |
| Glucose | Negative | 454 (13.8%) | 454 (13.8%) |
| Ketones | Negative | 611 (18.6%) | 611 (18.6%) |
| Bilirubin | Negative | 233 (7.1%) | 233 (7.1%) |

FI_Lab: frailty index based on physiological and laboratory tests; APTT: activated partial thromboplastin time; PH: potential of hydrogen; PO2: partial oxygen pressure; PCO2: partial carbon dioxide pressure; SD: standard deviation; IQR: inter-quartile range

### Supplementary Table 3. Included patients’ characteristics according to survival status as of hospital discharge

| Variable | Total (n=3,273) | Survived (*N*=2,714) | Did not survive (*N*=559) | *P*-value |
| --- | --- | --- | --- | --- |
| ***Demographic*** |  |  |  |  |
| Age, years, median (Q1, Q3) | 74.79 (64.28, 83.25) | 73.96 (63.49, 83.02) | 77.74 (68.65, 84.27) | < 0.001 |
| Sex, *N* (%) |  |  |  | 0.180 |
| Male | 1,820 (56) | 1,524 (56) | 296 (53) |  |
| Ethnicity, *N* (%) |  |  |  | 0.002 |
| White | 2,164 (66) | 1,821 (67) | 343 (61) |  |
| Black | 288 (9) | 245 (9) | 43 (8) |  |
| Other | 821 (25) | 648 (24) | 173 (31) |  |
| Admission location, *N* (%) |  |  |  | 0.041 |
| Emergency department | 1,195 (37) | 982 (36) | 213 (38) |  |
| Other hospital | 1,977 (60) | 1,642 (61) | 335 (60) |  |
| Operating room | 57 (2) | 55 (2) | 2 (0) |  |
| Other | 44 (1) | 35 (1) | 9 (2) |  |
| ***Comorbidities*** |  |  |  |  |
| Obesity, *N* (%) | 507 (15) | 439 (16) | 68 (12) | 0.020 |
| Hypertension, *N* (%) | 984 (30) | 857 (32) | 127 (23) | < 0.001 |
| Diabetes, *N* (%) | 1,313 (40) | 1,090 (40) | 223 (40) | 0.943 |
| AKI^a^, *N* (%) | 2,707 (83) | 2,182 (80) | 525 (94) | < 0.001 |
| CCI, points, median (Q1, Q3) | 7 (6, 9) | 7 (6, 9) | 8 (7, 10) | < 0.001 |
| ***ICU admission*** |  |  |  |  |
| SAPSII score, median (Q1, Q3) | 41 (33, 51) | 39 (32, 48) | 52 (43, 62) | < 0.001 |
| SOFA score, median (Q1, Q3) | 6 (4, 9) | 6 (4, 9) | 10 (7, 13) | < 0.001 |
| APSIII score, median (Q1, Q3) | 53 (40, 72) | 49 (38, 65) | 82 (61, 102) | < 0.001 |
| LODS score, median (Q1, Q3) | 6 (4, 9) | 5 (3, 8) | 9 (7, 12) | < 0.001 |
| OASIS score, median (Q1, Q3) | 35 (28, 42) | 33 (27, 40) | 44 (36.5, 50) | < 0.001 |
| SIRS score, median (Q1, Q3) | 3 (2, 3) | 3 (2, 3) | 3 (2, 3) | < 0.001 |
| FI_Lab, median (Q1, Q3) | 0.42 (0.33, 0.48) | 0.39 (0.30, 0.48) | 0.52 (0.42, 0.58) | < 0.001 |

^a^AKI was defined according to KDIGO guidelines as a ≥0.3 mg/dL increase in serum creatinine from baseline within 48 h, an increase of at least 1.5 times the baseline level recorded in the preceding 7 days, or a urine output of <0.5 ml/kg/hour for 6 hours or more.

CCI: Charlson comorbidity index; ICU: intensive care unit; SAPS II: simplified acute physiological score II; SOFA: sequential organ failure assessment; APS III: acute physiology score III; LODS: logistic organ dysfunction system; SIRS: systemic inflammatory response syndrome; OASIS: Oxford acute severity of illness; FI_Lab: frailty index based on both physiological and laboratory tests

### Supplementary Table 4. The association between FI_Lab and incidence of AKI and RRT use in patients with AKI

| **Categories** | **Events (%)** | **Model 1** | **Model 2** | **Model 3** |
| --- | --- | --- | --- | --- |
| **Incidence of AKI** | | **OR (95% CI)** | **OR (95% CI)** | **OR (95% CI)** |
| Continuous variable  (per 0.01-score) | | 1.048 (1.039-1.058) | 1.049 (1.040-1.058) | 1.015 (1.005-1.026) |
| Non-frail | 445 (63.1) | Ref. | Ref. | Ref. |
| Pre-frail | 564 (73.2) | 1.600 (1.641-3.027) | 1.606 (1.287-2.004) | 1.156 (0.908-1.471) |
| Frail | 564 (85.8) | 3.543 (2.712-4.630) | 3.603 (2.755-4.712) | 1.403 (1.031-1.909) |
| *P* for trend |  | <0.001 | <0.001 | 0.031 |
| **RRT use in patients with AKI** | | **HR (95% CI)** | **HR (95% CI)** | **HR (95% CI)** |
| Continuous variable  (per 0.01-score) | | 1.070 (1.060-1.080) | 1.060 (1.050-1.070) | 1.020 (1.010-1.030) |
| Non-frail | 21 (2.7) | Ref. | Ref. | Ref. |
| Pre-frail | 94 (9.4) | 3.120 (1.950-4.990) | 3.060 (1.910-4.90) | 2.070 (1.290-3.320) |
| Frail | 226 (23.5) | 7.480 (4.770-11.70) | 7.190 (4.580-11.30) | 2.890 (1.790-4.650) |
| *P* for trend |  | <0.001 | <0.001 | <0.001 |

Model 1: unadjusted.

Model 2: adjusted for age, sex, ethnicity and admission location

Model 3: adjusted for age, sex, ethnicity, admission location, SOFA score and CCI points

FI_Lab: frailty index based on physiological and laboratory tests; AKI was defined as occurring 24 hours after admission to the ICU according to the KDIGO guidelines. RRT: renal replacement therapy used in patients with AKI; SOFA: sequential organ failure assessment; CCI: Charlson comorbidity index

### Supplementary Table 5. Sensitivity analysis for the association between FI_Lab and in-hospital mortality adjusted for APS III and SAPS II scores in the multivariable model

| **Categories** | **Events (%)** | **Model 1** | **Model 2** | **Model 3** | **Model 4** |
| --- | --- | --- | --- | --- | --- |
| **In-hospital mortality** | | **OR (95% CI)** | **OR (95% CI)** | **OR (95% CI)** | **OR (95% CI)** |
| Continuous variable  (per 0.01-score) | | 1.071 (1.062-1.08) | 1.074 (1.066-1.083) | 1.029 (1.019-1.040) | 1.046 (1.037-1.056) |
| Categorical variable | |  |  |  |  |
| Non-frail | 63 (6.1) | Ref. | Ref. | Ref. | Ref. |
| Pre-frail | 153 (12.7) | 2.229 (1.641-3.027) | 2.268 (1.668-3.084) | 1.382 (0.996-1.916) | 1.667 (1.214-2.290) |
| Frail | 343 (32.5) | 7.326 (5.505-9.747) | 7.848 (5.882-10.471) | 2.298 (1.656-3.188) | 3.694 (2.703-5.049) |
| *P* for trend |  | <0.001 | <0.001 | <0.001 | <0.001 |

Model 1: unadjusted

Model 2: adjusted for age, sex, ethnicity and admission location

Model 3: adjusted for age, sex, ethnicity, admission location, APS III scores and CCI points

Model 4: adjusted for age, sex, ethnicity, admission location, SAPS II scores and CCI points

FI_Lab: frailty index based on physiological and laboratory tests; CCI: Charlson comorbidity index; APSIII: acute physiology score III; SAPSII: simplified acute physiological score II

| **Models** | **AUC (95%CI)** | **Models** | **AUC (95%CI)** | **Δ AUC** | ***P*-value for Δ AUC** | **IDI (95%CI)** | ***P*-value** | **NRI (95%CI)** | ***P*-value** |
| --- | --- | --- | --- | --- | --- | --- | --- | --- | --- |
| **In-hospital mortality** | |  |  |  |  |  |  |  |  |
| SOFA | 0.74 (0.71-0.76) | +FI_Lab | 0.76 (0.74-0.78) | 0.025 | <0.001 | 0.025(0.019 - 0.030) | <0.001 | 0.426(0.339 - 0.513) | <0.001 |
| APSIII | 0.80 (0.78-0.82) | +FI_Lab | 0.80 (0.78-0.82) | 0.007 | 0.030 | 0.009(0.005 - 0.013) | <0.001 | 0.360(0.272 - 0.449) | <0.001 |
| SAPSII | 0.75 (0.73-0.77) | +FI_Lab | 0.77 (0.75-0.79) | 0.021 | <0.001 | 0.028(0.022 - 0.034) | <0.001 | 0.454(0.366 - 0.543) | <0.001 |
| LODS | 0.78 (0.76-0.80) | +FI_Lab | 0.79 (0.77-0.81) | 0.016 | <0.001 | 0.020(0.014 - 0.026) | <0.001 | 0.505(0.418 - 0.592) | <0.001 |
| OASIS | 0.76 (0.74-0.78) | +FI_Lab | 0.79 (0.77-0.81) | 0.029 | <0.001 | 0.031(0.024 - 0.038) | <0.001 | 0.505(0.417 - 0.593) | <0.001 |
| SIRS | 0.59 (0.57-0.61) | +FI_Lab | 0.72 (0.69-0.74) | 0.127 | <0.001 | 0.071(0.062 - 0.080) | <0.001 | 0.656(0.569 - 0.743) | <0.001 |
| **1-year mortality** | |  |  |  |  |  |  |  |  |
| SOFA | 0.54 (0.52-0.57) | +FI_Lab | 0.61 (0.58-0.63) | 0.067 | <0.001 | 0.027(0.021 - 0.033) | <0.001 | 0.383(0.299 - 0.466) | <0.001 |
| APSIII | 0.62 (0.60-0.64) | +FI_Lab | 0.63 (0.61-0.65) | 0.011 | 0.145 | 0.013(0.009 - 0.018) | <0.001 | 0.272(0.188 - 0.356) | <0.001 |
| SAPSII | 0.64 (0.61-0.66) | +FI_Lab | 0.65 (0.63-0.67) | 0.011 | 0.078 | 0.012(0.008 - 0.016) | <0.001 | 0.243(0.158 - 0.328) | <0.001 |
| LODS | 0.59 (0.57-0.61) | +FI_Lab | 0.62 (0.60-0.65) | 0.033 | <0.001 | 0.018(0.013 - 0.024) | <0.001 | 0.283(0.200 - 0.367) | <0.001 |
| OASIS | 0.58 (0.56-0.61) | +FI_Lab | 0.63 (0.60-0.65) | 0.049 | <0.001 | 0.019(0.014 - 0.024) | <0.001 | 0.278(0.194 - 0.362) | <0.001 |
| SIRS | 0.50 (0.48-0.52) | +FI_Lab | 0.62 (0.59-0.64) | 0.115 | <0.001 | 0.032(0.025 - 0.038) | <0.001 | 0.321(0.236 - 0.405) | <0.001 |
| **Incidence of AKI** | |  |  |  |  |  |  |  |  |
| SOFA | 0.74 (0.72-0.76) | +FI_Lab | 0.74 (0.72-0.76) | 0.003 | 0.126 | 0.002(0.000 - 0.004) | 0.086 | 0.146(0.050 - 0.242) | 0.003 |
| APSIII | 0.73 (0.70-0.75) | +FI_Lab | 0.73 (0.70-0.75) | 0.000 | 0.603 | 0.000(0.000 - 0.002) | 0.238 | 0.075 (-0.022-0.171) | 0.129 |
| SAPSII | 0.71 (0.68-0.73) | +FI_Lab | 0.71 (0.69-0.74) | 0.004 | 0.230 | 0.007(0.003 - 0.011) | <0.001 | 0.163(0.066 - 0.259) | <0.001 |
| LODS | 0.74 (0.72-0.77) | +FI_Lab | 0.75 (0.72-0.77) | 0.000 | 0.302 | 0.001(0.000 - 0.003) | 0.175 | 0.102(0.005 - 0.198) | 0.039 |
| OASIS | 0.75 (0.73-0.77) | +FI_Lab | 0.75 (0.73-0.77) | 0.003 | 0.241 | 0.005(0.001 - 0.008) | 0.009 | 0.128(0.031 - 0.224) | 0.009 |
| SIRS | 0.57 (0.54-0.59) | +FI_Lab | 0.64 (0.61-0.66) | 0.073 | <0.001 | 0.034(0.026 - 0.042) | <0.001 | 0.334(0.239- 0.429) | <0.001 |
| **RRT use in patients with AKI** | | |  |  |  |  |  |  |  |
| SOFA | 0.80 (0.77-0.82) | +FI_Lab | 0.81 (0.79-0.83) | 0.014 | 0.002 | 0.012(0.006 - 0.017) | <0.001 | 0.421(0.311 - 0.531) | <0.001 |
| APSIII | 0.79 (0.77-0.81) | +FI_Lab | 0.80 (0.78.0.82) | 0.009 | 0.103 | 0.013(0.007 - 0.018) | <0.001 | 0.500(0.390 - 0.610) | <0.001 |
| SAPSII | 0.73 (0.71-0.76) | +FI_Lab | 0.77 (0.74-0.79) | 0.032 | <0.001 | 0.030(0.023 - 0.037) | <0.001 | 0.569(0.460 - 0.679) | <0.001 |
| LODS | 0.78 (0.76-0.81) | +FI_Lab | 0.80 (0.78-0.82) | 0.017 | 0.002 | 0.021(0.014 - 0.027) | <0.001 | 0.612(0.505 - 0.718) | <0.001 |
| OASIS | 0.70 (0.67-0.73) | +FI_Lab | 0.76 (0.73-0.78) | 0.054 | <0.001 | 0.038(0.030 - 0.045) | <0.001 | 0.626(0.520 - 0.733) | <0.001 |
| SIRS | 0.57 (0.54-0.60) | +FI_Lab | 0.71 (0.69-0.74) | 0.146 | <0.001 | 0.061(0.053 - 0.070) | <0.001 | 0.704(0.597 - 0.811) | <0.001 |

### Supplementary Table 6. Sensitivity analysis for the incremental FI_Lab value as a categorical variable for outcomes

FI_Lab: frailty index based on physiological and laboratory tests; IDI: integrated discrimination improvement; NRI: net reclassification improvement; AKI was defined as occurring 24 hours after admission to the ICU, as according to the KDIGO guidelines. RRT: renal replacement therapy used in patients with AKI; SOFA: sequential organ failure assessment; APS III: acute physiology score III; SAPS II: simplified acute physiological score II; LODS: logistic organ dysfunction system; OASIS: Oxford acute severity of illness score; SIRS: systemic inflammatory response syndrome

### Supplementary Table 7. Incremental FI_Lab value as a continuous variable for outcomes

| **Models** | **AUC (95%CI)** | **Models** | **AUC (95%CI)** | | **Δ AUC** | | ***P*-value for Δ AUC** | | | **IDI (95%CI)** | | ***P*-value** | | | | **NRI (95%CI)** | ***P*-value** |
| --- | --- | --- | --- | --- | --- | --- | --- | --- | --- | --- | --- | --- | --- | --- | --- | --- | --- |
| **In-hospital mortality** | |  |  |  | | |  | | | |  |  | | | |  |  |
| SOFA | 0.74 (0.71-0.76) | +FI_Lab | 0.76 (0.74-0.79) | | | 0.027 | <0.001 | 0.030 (0.023 - 0.038) | | | | | <0.001 | 0.3847 (0.295 - 0.474) | | | <0.001 |
| APSIII | 0.80 (0.78-0.82) | +FI_Lab | 0.81 (0.79-0.82) | | | 0.008 | 0.009 | 0.012 (0.006 - 0.017) | | | | | <0.001 | 0.2719 (0.181 - 0.362) | | | <0.001 |
| SAPSII | 0.75 (0.73-0.77) | +FI_Lab | 0.78 (0.76-0.80) | | | 0.024 | <0.001 | 0.032 (0.025 - 0.040) | | | | | <0.001 | 0.4218 (0.332 - 0.511) | | | <0.001 |
| LODS | 0.78 (0.76-0.80) | +FI_Lab | 0.81 (0.79-0.82) | | | 0.008 | 0.009 | 0.026 (0.018 - 0.033) | | | | | <0.001 | 0.4316 (0.342 - 0.521) | | | <0.001 |
| OASIS | 0.76 (0.74-0.78) | +FI_Lab | 0.79 (0.77-0.81) | | | 0.034 | <0.001 | 0.039 (0.030 - 0.048) | | | | | <0.001 | 0.4111 (0.322 - 0.501) | | | <0.001 |
| SIRS | 0.59 (0.57-0.61) | +FI_Lab | 0.73 (0.71-0.76) | | | 0.143 | <0.001 | 0.090 (0.078 - 0.102) | | | | | <0.001 | 0.6545 (0.567 - 0.742) | | | <0.001 |
| **1-year mortality** | |  |  |  | | |  |  | | | | |  | | |  |  |
| SOFA | 0.54 (0.52-0.57) | +FI_Lab | 0.63 (0.60-0.65) | | | 0.086 | <0.001 | | 0.033 (0.026 - 0.040) | | | <0.001 | | | 0.356 (0.272 - 0.440) | | <0.001 |
| APSIII | 0.62 (0.60-0.64) | +FI_Lab | 0.63 (0.61-0.66) | | | 0.014 | 0.088 | | 0.016 (0.011 - 0.021) | | | <0.001 | | | 0.290 (0.206 - 0.375) | | <0.001 |
| SAPSII | 0.64 (0.61-0.66) | +FI_Lab | 0.65 (0.63-0.67) | | | 0.015 | 0.028 | | 0.014 (0.009 - 0.018) | | | <0.001 | | | 0.280 (0.196 - 0.365) | | <0.001 |
| LODS | 0.59 (0.57-0.61) | +FI_Lab | 0.63 (0.60-0.65) | | | 0.038 | <0.001 | | 0.022 (0.017 - 0.028) | | | <0.001 | | | 0.297 (0.213 - 0.382) | | <0.001 |
| OASIS | 0.58 (0.56-0.61) | +FI_Lab | 0.63 (0.61-0.65) | | | 0.047 | <0.001 | | 0.023 (0.017 - 0.029) | | | <0.001 | | | 0.280 (0.196 - 0.365) | | <0.001 |
| SIRS | 0.50 (0.48-0.52) | +FI_Lab | 0.63 (0.61-0.65) | | | 0.131 | <0.001 | | 0.038 (0.030 - 0.045) | | | <0.001 | | | 0.357 (0.273 - 0.441) | | <0.001 |
| **Incidence of AKI** | |  |  | | |  |  | |  | | |  | | |  | |  |
| SOFA | 0.74 (0.72-0.76) | +FI_Lab | 0.74 (0.72-0.77) | | | 0.005 | 0.085 | | 0.003 (0.000 - 0.006) | | | 0.028 | | | 0.045 (-0.052 - 0.141) | | 0.364 |
| APSIII | 0.73 (0.70-0.75) | +FI_Lab | 0.73 (0.70-0.75) | | | 0.001 | 0.461 | | 0.003 (0.000 - 0.005) | | | 0.032 | | | 0.006 (-0.103 - 0.090) | | 0.901 |
| SAPSII | 0.71 (0.68-0.73) | +FI_Lab | 0.72 (0.69-0.74) | | | 0.007 | 0.106 | | 0.010 (0.005 - 0.015) | | | <0.001 | | | 0.147 (0.050 - 0.243) | | 0.003 |
| LODS | 0.74 (0.72-0.77) | +FI_Lab | 0.75 (0.72-0.77) | | | 0.003 | 0.221 | | 0.003 (0.000 - 0.006) | | | 0.041 | | | 0.037 ( -0.059 -0.133) | | 0.452 |
| OASIS | 0.75 (0.73-0.77) | +FI_Lab | 0.75 (0.73-0.78) | | | 0.005 | 0.145 | | 0.009 (0.004 - 0.013) | | | <0.001 | | | 0.142 (0.045 - 0.238) | | <0.001 |
| SIRS | 0.57 (0.54-0.59) | +FI_Lab | 0.65 (0.63-0.68) | | | 0.085 | <0.001 | | 0.047 (0.037 - 0.056) | | | <0.001 | | | 0.417 (0.324 - 0.511) | | <0.001 |
| **RRT use in patients with AKI** | | |  | | |  |  | |  | | |  | | |  | |  |
| SOFA | 0.80 (0.77-0.82) | +FI_Lab | 0.81 (0.79-0.83) | | | 0.015 | <0.001 | | 0.012 (0.006 - 0.019) | | | <0.001 | | | 0.302 (0.190 - 0.414) | | <0.001 |
| APSIII | 0.79 (0.77-0.81) | +FI_Lab | 0.80 (0.78-0.83) | | | 0.013 | 0.011 | | 0.016 (0.008 - 0.024) | | | <0.001 | | | 0.407 (0.295 - 0.519) | | <0.001 |
| SAPSII | 0.73 (0.71-0.76) | +FI_Lab | 0.77 (0.75-0.80) | | | 0.038 | <0.001 | | 0.035 (0.026 - 0.044) | | | <0.001 | | | 0.490 (0.380 - 0.601) | | <0.001 |
| LODS | 0.78 (0.76-0.81) | +FI_Lab | 0.80 (0.78-0.83) | | | 0.022 | <0.001 | | 0.025 (0.016 - 0.034) | | | <0.001 | | | 0.456 (0.345 - 0.568) | | <0.001 |
| OASIS | 0.70 (0.67-0.73) | +FI_Lab | 0.77 (0.74-0.79) | | | 0.063 | <0.001 | | 0.048 (0.038 - 0.058) | | | <0.001 | | | 0.543 (0.434 - 0.653) | | <0.001 |
| SIRS | 0.57 (0.54-0.60) | +FI_Lab | 0.74 (0.71-0.77) | | | 0.173 | <0.001 | | 0.732 (0.628 - 0.837) | | | <0.001 | | | 0.079 (0.066 - 0.092) | | <0.001 |

FI_Lab: frailty index based on physiological and laboratory tests; IDI: integrated discrimination improvement; NRI: net reclassification improvement; AKI was defined as occurring 24 hours after admission to the ICU, as according to the KDIGO guidelines. RRT: renal replacement therapy used in patients with AKI; SOFA: sequential organ failure assessment; APS III: acute physiology score III; SAPS II: simplified acute physiological score II; LODS: logistic organ dysfunction system; OASIS: Oxford acute severity of illness; SIRS: systemic inflammatory response syndrome

| **Items** | **AUC**^a^ **(95%CI)** | ***P –* Value**^b^ |
| --- | --- | --- |
| **Vital signs** |  |  |
| Systolic blood pressure (mm Hg) | 0.49 (0.48-0.51) | <0.001 |
| Diastolic blood pressure (mm Hg) | 0.55 (0.53-0.57) | <0.001 |
| Heart rate (bpm) | 0.55 (0.53-0.57) | <0.001 |
| **Venous blood samples** |  |  |
| White cell count (×10^3^/μL) | 0.58 (0.56-0.60) | <0.001 |
| Platelet count (×10^9^/L) | 0.55 (0.52-0.57) | <0.001 |
| Hemoglobin (g/dL) | 0.51 (0.50-0.53) | <0.001 |
| Red blood cell distribution width (%) | 0.60 (0.57-0.62) | <0.001 |
| Total bilirubin (mg/dL) | 0.56 (0.54-0.58) | <0.001 |
| Alanine transaminase (Units/L) | 0.56 (0.54-0.59) | <0.001 |
| Albumin (g/dL) | 0.61 (0.59-0.63) | <0.001 |
| Alkaline phosphatase (Units/L) | 0.55 (0.53-0.58) | <0.001 |
| Lactate dehydrogenase (Units/L) | 0.61 (0.59-0.63) | <0.001 |
| Urea nitrogen (mg/dL) | 0.57 (0.55-0.59) | <0.001 |
| Creatinine (mg/dL) | 0.59 (0.57-0.61) | <0.001 |
| Glucose (mg/dL) | 0.51 (0.49-0.53) | <0.001 |
| Potassium (mmol/L) | 0.52 (0.51-0.54) | <0.001 |
| Sodium (mmol/L) | 0.54 (0.52-0.56) | <0.001 |
| Calcium (mg/dL) | 0.56 (0.54-0.58) | <0.001 |
| Phosphorus (mg/dL) | 0.58 (0.56-0.60) | <0.001 |
| Prothrombin time (s) | 0.55 (0.53-0.56) | <0.001 |
| International normalized ratio | 0.54 (0.53-0.56) | <0.001 |
| APTT (s) | 0.54 (0.52-0.56) | <0.001 |
| Fibrinogen (mg/dL) | 0.58 (0.55-0.62) | <0.001 |
| **Arterial blood gas samples** |  |  |
| PH | 0.59 (0.56-0.61) | <0.001 |
| PO_2_ (mm Hg) | 0.47 (0.45-0.49) | <0.001 |
| PCO_2_ (mm Hg) | 0.55 (0.53-0.57) | <0.001 |
| Lactate (mmol/L) | 0.60 (0.58-0.63) | <0.001 |
| **Urine sample** |  |  |
| Leucocytes | 0.54 (0.51-0.56) | <0.001 |
| Erythrocytes | 0.54 (0.51-0.56) | <0.001 |
| Protein | 0.58 (0.56-0.60) | <0.001 |
| Glucose | 0.53 (0.51-0.55) | <0.001 |
| Ketones | 0.53 (0.51-0.55) | <0.001 |
| Bilirubin | 0.54 (0.52-0.55) | <0.001 |

### Supplementary Table 8. Predictive accuracy of each item in the FI_Lab for in-hospital mortality.

^a^AUC was adjusted for age, sex. FI-Lab, ^b^*P* Value, compared with FI_Lab by DeLong’s test. FI_Lab, frailty index based on physiological and laboratory tests; APTT, activated partial thromboplastin time; PH, potential of hydrogen; PO_2_, partial pressure of oxygen; PCO_2_, partial pressure of carbon dioxide.

| **Items** | **AUC**^a^ **(95%CI)** | ***P –* Value**^b^ |
| --- | --- | --- |
| **Vital signs** |  |  |
| Systolic blood pressure (mm Hg) | 0.50 (0.49-0.51) | <0.001 |
| Diastolic blood pressure (mm Hg) | 0.53 (0.51-0.55) | <0.001 |
| Heart rate (bpm) | 0.49 (0.47-0.51) | <0.001 |
| **Venous blood samples** |  |  |
| White cell count (×10^3^/μL) | 0.49 (0.47-0.52) | <0.001 |
| Platelet count (×10^9^/L) | 0.52 (0.50-0.54) | <0.001 |
| Hemoglobin (g/dL) | 0.51 (0.50-0.53) | <0.001 |
| Red blood cell distribution width (%) | 0.60 (0.58-0.62) | <0.001 |
| Total bilirubin (mg/dL) | 0.53 (0.51-0.54) | <0.001 |
| Alanine transaminase (Units/L) | 0.50 (0.48-0.52) | <0.001 |
| Albumin (g/dL) | 0.58 (0.56-0.61) | <0.001 |
| Alkaline phosphatase (Units/L) | 0.53 (0.51-0.55) | <0.001 |
| Lactate dehydrogenase (Units/L) | 0.53 (0.50-0.56) | <0.001 |
| Urea nitrogen (mg/dL) | 0.59 (0.58-0.61) | <0.001 |
| Creatinine (mg/dL) | 0.60 (0.57-0.62) | <0.001 |
| Glucose (mg/dL) | 0.51 (0.49-0.53) | <0.001 |
| Potassium (mmol/L) | 0.52 (0.50-0.53) | <0.001 |
| Sodium (mmol/L) | 0.53 (0.51-0.55) | <0.001 |
| Calcium (mg/dL) | 0.51 (0.49-0.53) | <0.001 |
| Phosphorus (mg/dL) | 0.54 (0.52-0.56) | <0.001 |
| Prothrombin time (s) | 0.52 (0.51-0.54) | <0.001 |
| International normalized ratio | 0.52 (0.50-0.53) | <0.001 |
| APTT (s) | 0.52 (0.49-0.54) | <0.001 |
| Fibrinogen (mg/dL) | 0.58 (0.55-0.62) | <0.001 |
| **Arterial blood gas samples** |  |  |
| PH | 0.56 (0.53, 0.58) | <0.001 |
| PO_2_ (mm Hg) | 0.48 (0.46-0.50) | <0.001 |
| PCO_2_ (mm Hg) | 0.56 (0.53-0.58) | <0.001 |
| Lactate (mmol/L) | 0.50 (0.47-0.52) | <0.001 |
| **Urine sample** |  |  |
| Leucocytes | 0.58 (0.58, 0.61) | 0.004 |
| Erythrocytes | 0.58 (0.56, 0.61) | 0.004 |
| Protein | 0.55 (0.53, 0.56) | <0.001 |
| Glucose | 0.51 (0.49, 0.53) | <0.001 |
| Ketones | 0.51 (0.49, 0.53) | <0.001 |
| Bilirubin | 0.51 (0.50, 0.52) | <0.001 |

### Supplementary Table 9. Predictive Ability of Each Item in the FI_Lab for 1-year Mortality.

^a^AUC was adjusted adjusted for age, sex . FI-Lab, ^b^*P* Value, compared with FI_Lab by DeLong’s test. FI_Lab, frailty index based on physiological and laboratory tests; APTT, activated partial thromboplastin time; PH, potential of hydrogen; PO_2_, partial pressure of oxygen; PCO_2_, partial pressure of carbon dioxide.
